# Determinants of malaria among under-five children in Ethiopia: Bayesian multilevel analysis

**DOI:** 10.1186/s12889-020-09560-1

**Published:** 2020-09-29

**Authors:** Setognal Birara Aychiluhm, Kassahun Alemu Gelaye, Dessie Abebaw Angaw, Getachew Asfaw Dagne, Abay Woday Tadesse, Adugna Abera, Dereje Dillu

**Affiliations:** 1 Department of Public Health, College of Medicine and Health Sciences, Samara University, Samara, Ethiopia; 2 Department of Epidemiology and Biostatistics, Institute of Public Health, College of Medicine and Health Sciences, University of Gondar, Gondar, Ethiopia; 3 College of Public Health, University of South Florida, Florida, USA; 4 Ethiopian Public Health Institute, Addis Ababa, Ethiopia; 5 Ethiopian Ministry of Health, Addis Ababa, Ethiopia

**Keywords:** Malaria microscopy test, Bayesian multilevel logistic regression, Ethiopia

## Abstract

**Background:**

In Ethiopia, malaria is one of the public health problems, and it is still among the ten top leading causes of morbidity and mortality among under-five children.

However, the studies conducted in the country have been inconclusive and inconsistent. Thus, this study aimed to assess factors associated with malaria among under-five children in Ethiopia.

**Methods:**

We retrieved secondary data from the malaria indicator survey data collected from September 30 to December 10, 2015, in Ethiopia. A total of 8301 under-five-year-old children who had microscopy test results were included in the study. Bayesian multilevel logistic regression models were fitted and Markov chain Monte Carlo simulation was used to estimate the model parameters using Gibbs sampling. Adjusted Odd Ratio with 95% credible interval in the multivariable model was used to select variables that have a significant association with malaria.

**Results:**

In this study, sleeping under the insecticide-treated bed nets during bed time (ITN) [AOR 0.58,95% CI, 0.31–0.97)], having 2 and more ITN for the household [AOR 0.43, (95% CI, 0.17–0.88)], have radio [AOR 0.41, (95% CI, 0.19–0.78)], have television [AOR 0.19, (95% CI, 0.01–0.89)] and altitude [AOR 0.05, (95% CI, 0.01–0.13)] were the predictors of malaria among under-five children.

**Conclusions:**

The study revealed that sleeping under ITN, having two and more ITN for the household, altitude, availability of radio, and television were the predictors of malaria among under-five children in Ethiopia. Thus, the government should strengthen the availability and utilization of ITN to halt under-five mortality due to malaria.

## Background

Malaria is one of the most public health problems worldwide, in which 300 to 500 million cases and more than one million deaths were reported in 2018. Of these deaths, 90% were reported from Sub-Saharan African countries [1, 2]. This is equivalent to one child in sub-Saharan Africa dying of malaria every 2 min, and it is one of the fourth leading cause of death of children under the age of 5 years in developing countries in general [1, 3].

In Ethiopia, 68% of the areas are endemic for malaria, and 60% of the country population are prone for infection of malaria [4]. Transmission of malaria mainly depends on the temperature, humidity, and rainfall [5]. In addition, its transmission is also determined by socioeconomic conditions, demographic factors, community-related factors, knowledge, and access to malaria prevention modalities [6].

After the increased utilization of indoor residual spray (IRS), Artemisinin-based combination therapy (ACT), and insecticide-treated bed nets (ITNs), deaths and admissions due to malaria among under-five children decreased by 81 and 73% respectively [7].

Nevertheless, still malaria is one of the ten top leading causes of morbidity and mortality among under-five children in Ethiopia [8]. Accordingly, morbidity and mortality rates of the disease blow up during epidemics time [9].

In the last decades, numerous strategies, policies, and malaria elimination programs had been implemented at the global and national levels [10–12]. As a result, over 6.2 million malaria deaths were prevented between 2000 and 2015 in sub-Saharan African countries [11]. Thus, despite the significant decline in the burden of malaria, the disease is still one of the major public health concerns in Ethiopia [13]. Moreover, the magnitude of malaria infection among under-five children is too high which ranges from 16 to 54% in Ethiopia [14–18].

Former studies conducted across the world have identified determinants of malaria among under-five children. Some of the determinants include; altitude of residence site, number of bed nets in the household, child sex, child age, living near to dam, housing conditions, presence of forest cover, and family size [15–17, 19–21]. Besides, there is still a need to investigate and identify the effect of these factors among under-five children in our country, Ethiopia, to allow an effective preparation of a national malaria prevention, control strategy, and intervention guidelines.

In Ethiopia, although some studies have been conducted to determine factors of malaria among under-five children, those previous studies [16, 17, 22–27] were limited (geographic area) and inconclusive (relatively small sample size) to show the determinants of malaria at the national level. Also, those previous studies did not include some community level factors like region, enumeration area, and altitude at national level. In this study, we have included those independent community-level variables, with a relatively large sample size that could help to make inference at a national level, besides we have also used a new statistical Bayesian multilevel analysis approach to assess the determinants of malaria infection. This Bayesian multilevel analysis approach is a powerful statistical method in medical and public health researches [28] that is done based on the data at hand and prior information that has been existed before.

## Methods and materials

### Study area

Ethiopia is an East African country with an estimated population of more than 100 million which makes it the second-most populous country in Africa [29]. Administratively, Ethiopia is divided into regions and regions are divided into zones, and zones, into administrative units called district (wereda). Each district is further subdivided into kebele that is the lowest administrative unit. Kebeles are also further subdivided into enumeration areas (EAs). Ethiopia, located within 3.30°–15°N, 33°–48°E in the northeastern part of Africa, and it has a total area of 1.1 million square kilometers. It’s topographic features ranges from mountains as high as Ras Dashen 4550 m (m) above sea level (ASL)—to 110 m below sea level in the Afar Depression [4].

Around two-thirds of the country’s territory is favorable for malaria transmission, with malaria primarily associated with altitude and rainfall. Approximately 60% of Ethiopia’s population lives in a malarious area. The highest period of malaria incidence occurs from September to December and from March to May in most parts of the country. The proportion of the population consisting of children under-5 years of age and pregnant women was estimated to be 14.6% and 3.3%, respectively [4].

### Data source, study design, sampling procedure, and sample size

This study used cross-sectional survey data from a secondary source extracted from the Ethiopian Malaria Indicator Survey (EMIS), 2015. The EMIS 2015 was the third survey conducted in Ethiopia, a nationwide sample of 13,875 households from 555 EAs was selected.

The EMIS 2015 was used a two-stage cluster sampling methodology. 555EAs were selected in the first stage. Then a complete mapping and listing of all households in the selected EAs were conducted and 25 households were randomly selected for a total of 13, 875 households. Also, the survey involved testing for anemia and malaria among under-five children in all selected households [4].

Since the microscopic examination is the gold standard for the diagnosis of malaria, for this study, children were considered as malaria positive or negative based on theresult of this test only. In this study, we included all available relevant data for children under-5 years of age from the EMIS 2015. The sample size for this study was those all under-five children who were tested for malaria microscopy test. Thus, the number of children whose data were used in this study was 8301.

### Study variables

#### Dependent variable

Malaria microscopy test result among under-five children (Yes/No). The dependent variable was the malaria microscopy test result which was dichotomized into **Yes** if the test is positive for *Plasmodium falciparum*, *Plasmodium vivax*, and mixed infection (both falciparum and vivax) and **No** if the test negative for all species.

#### Independent variable

The determinants of malaria among under-five children were grouped into individual-level and community-level determinants.

Individual-related predictors include; the age of a child, sex of a child, household insecticide-treated net (ITN) ownership, the household status of indoor residual spraying (IRS), utilization of nets, number of nets in the household, availability of electricity, housing conditions of the household (floor materials, roof materials, and wall materials), water sources for drinking, time to get water, availability of television, availability of radio, toilet facilities, community-related predictors include; enumeration area, altitude, and region.

### Data management and statistical analysis

Sample allocation in the EMIS to different regions as well as urban and rural areas was not proportional. Thus, sample weights to the data were applied to estimate proportions and frequencies to adjust disproportionate sampling and non-response. Since the normality assumption was violated for continuous variables age and altitude, their median with interquartile range was reported in the descriptive analysis. Natural log transformation was applied for these variables before inclusion in the regression model to cure this problem.

The descriptive analysis was performed using both STATA (version 15) and R (version 3.5.2) statistical software and the inferential statistics were done by bayesian statistical software win BUGS (version1.4.3).

### Bayesian multilevel logistic regression model

In the usual classical statistics, the analysis of multilevel logistic regression model is based on estimating parameters through Maximum Likelihood Estimation (MLE) and given the asymptotic properties [30]. However, the Bayesian approach has an advantage over the classical approach in the estimation of the model parameters, which is conducted based on their posterior distribution [31].

The EMIS data has hierarchical nature and clustering effect expected in this hierarchical data nature. Therefore, to account for this clustering effect and to get unbiased parameter estimates bayesian multilevel logistic regression analysis was applied to identify determinants of malaria among under-five children in Ethiopia.

In this study, the basic data structure of the two-level logistic regression is a collection of J groups (enumeration areas) and within-group j *(j = 1,2,…, J)*, a random sample *n*_j_ of level-one units (individual children). The outcome variable is denoted by;

*Y*_*ij*_ = 1 if i^th^ children are in the j^th^ enumeration area is positive for microscopy test.

0 if the i^th^ children are in the j^th^ enumeration area is negative for microscopy test.

With probabilities, P_ij_ = 1/X_ij_, U_ij_) which is the probability of being positive for an i^th^ child (i = 1,2, …nj) from the j^th^ enumeration area. 1-P_ij_ is the probability of being negative for the i^th^ child (i = 1,2, …nj) from the j^th^ enumeration area. Therefore, the model is

$$ Logit\ {\left({Y}_{ij}\right)}_{-}\left( Xij\beta \right)+{U}_{0j\kern1.25em } where\ {U}_{0j}\sim N\left(0,{\sigma^2}_u\right) $$

Xij is the observed value of the predictor variable for a child i in an enumeration area *j* and U_0j_is a random effect.

### The convergence of the algorithm

In this study, Markov Chain Monte Carlo (MCMC) algorithm was carried out using the Bayesian statistical software Win BUGS version 1.4.3 [32]. The deviance information criterion (DIC) [33] was used to select the best fitted model. The empirical results from a given MCMC analysis are not deemed reliable until the chain has reached its stationary distribution. The term convergence of an MCMC algorithm denotes whether the algorithm has reached its equilibrium distribution or not. If the algorithm has reached its equilibrium distributions, then the generated sample derives from the true target distribution. Therefore, assessing the convergence of the algorithm is essential for producing results from the posterior distribution of interest.

To assess the convergence algorithm in our study, we used autocorrelation, time series plots, Gelman-Rubin statistic, and density plot. All the plots showed that the algorithm has reached its equilibrium (target) distribution for all parameters.

Summary statistics were carried out from the posterior distribution to describe the covariates and adjusted odds ratio (AOR) with corresponding 95% credible interval in the multilevel multivariable logistic regression model was used to select predictors of malaria among under-five children.

## Results

### Descriptive statistics of the respondents

From the overall respondents, approximately 51.19% of the total study participants were males.

The median age of children was 32 (interquartile range: 16–48 months). The median altitude of residence site was 1703 (interquartile range: 1259–1938 m) in the sampled households. A total of 3362(40.50%) of households had at least one insecticide-treated net and 4215(50.78%) of the children slept under insecticide-treated net the night before the survey took place. Less than half of 1866(22.48%) the interior walls of houses were sprayed. Furthermore, the majority of 6551(78.92) of respondents have radio in their household and only 568(6.85%) of the respondents have a television in their household (Table 1).

**Table 1:** Weighted Socioeconomic and Demographic Characteristics Associated with Malaria among under-five children in Ethiopia, 2015

Predictors	Category	Frequency (%)
Sex of Child	Male	4249 (51.19)
	Female	4052 (48.81)
Use of net	Yes	4086 (49.22)
	No	4215 (50.78)
Number of nets	None	4939 (59.50)
	One	1200 (14.46)
	Two &more	2162 (26.04)
Interior wall sprayed	Yes	1866 (22.48)
	No	6435 (77.52)
Have television	Yes	568 (6.85)
	No	7733 (93.15)
Have radio	Yes	6551 (78.92)
	No	1750 (21.08)
Have electricity	Yes	1316 (15.85)
	No	6985 (84.15)
Time to get water	< 30 min	5412 (65.20)
	≥30 min	2889 (34.80)
Roof materials	Corrugate	3658 (44.06)
	Stick and mud	1099 (13.24)
	Thatch	3544 (42.70)
Wall materials	Cement block	423 (5.09)
	Corrugated metal	105 (1.26)
	Mud/stick/wood	7773 (93.64)
Floor materials	Cement Floor	438 (5.28)
	Earth/Sand	7285 (87.77)
	Wood floor	578 (6.96)
Water source	Protected Water	1596 (19.23)
	Tap Water	3846 (46.33)
	Unprotected Water	2859 (34.44)
Toilet facilities	No facility	3616 (43.56)
	Pit latrine	3966 (47.78)
	Latrine with flush	719 (8.66)

### Malaria positivity in the study population

**Fig. 1:**
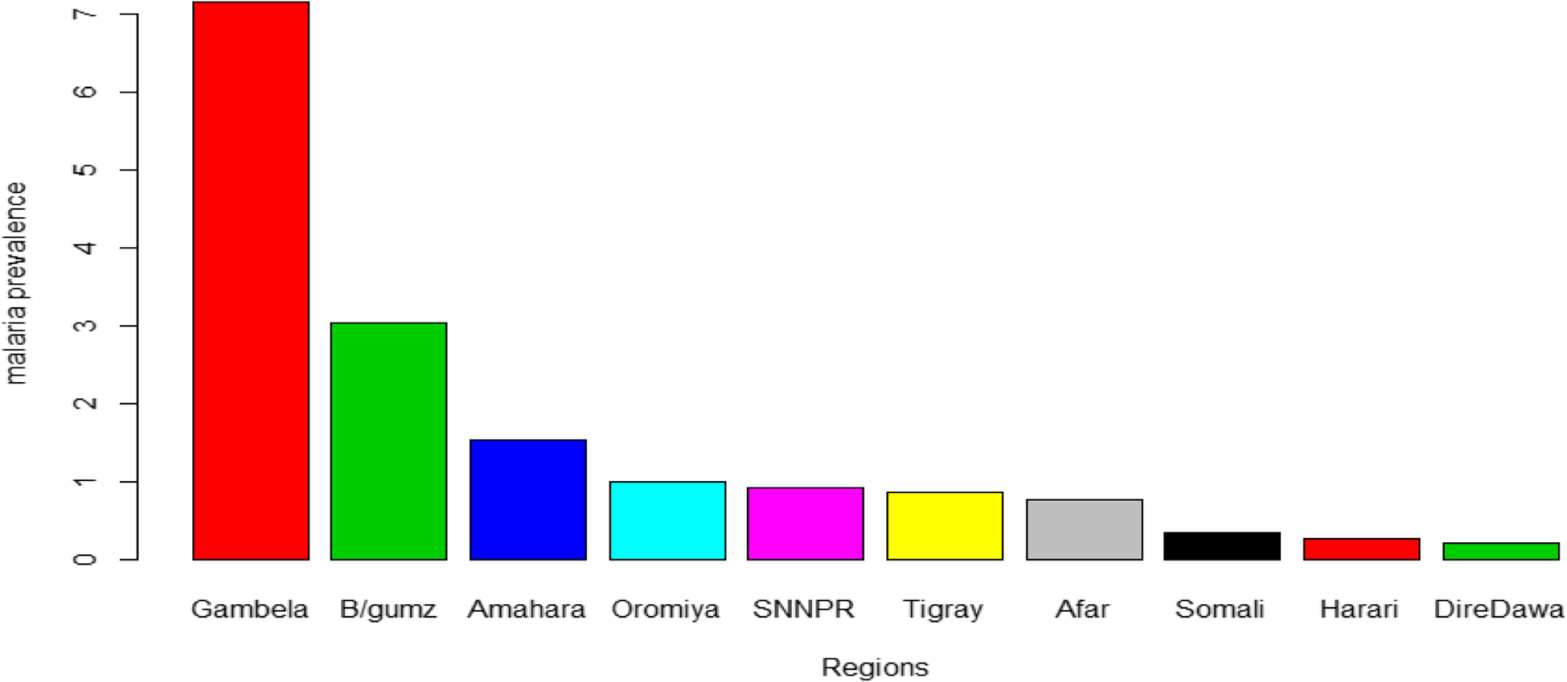
Prevalence of malaria under the age of 5 years old within each sampled region of Ethiopia, 2015

Among those who had a positive microscopy test result, the dominant plasmodium species were *Plasmodium falciparum* which accounts for 93.75% and followed by *Plasmodium vivax* which accounts 5.36%. In terms of regional variations, the highest prevalence of malaria was found among children from the Gambela region 5(7.14%) while the Benishangul gumuz region was the second-highest 41(3.04%) (Fig. 1).

### Result of empty (null) classical multilevel logistic regression model

In this null model, the random factor variance was 3.12 (95% CI: 2.18, 4.42). This variance value is greater than 0 and showed that there are EA differences in malaria status among under-five children in Ethiopia. The variance at the individual-level in logistic distributions equal to ∏^2^/3 (that is,3.29) [34–36].

Thus, the intra-enumeration area correlation coefficient (ICC) = 3.12/3.12 + 3.29 = 0.49, which showed that 49% of the overall variability in malaria status is through enumeration areas difference, with the rest 51% is due to the individual differences. Therefore, both the random factor variance and the ICC value suggested using multilevel logistic regression model to account for the EA differences in malaria status.

### Result of empty (null) Bayesian multilevel logistic regression model

The Bayesian null model showed that precision of the random factor was 0.19 with 95% credible interval of (0.03–0.58), showing EA differences in malaria status in the country. Since the variance estimate which is reciprocal of the precision (1/0.19 = 5.26) is greater than zero it indicates that there are EA differences in malaria status among under-five children in Ethiopia.

Since the unobserved heterogeneity have a logistic distribution with a variance at the individual-level equivalent to ∏^2^/3 (that is, 3.29) [34–36]. So, the ICC = 5.26/5.26 + 3.29 = 0.62, which implied that 62% of the total variability in malaria status among under-five children is because of differences across enumeration areas and 38% of the variability is accounted by individual differences (Table 2). Both the random factor variance and the ICC value proposed to apply bayesian multilevel logistic regression model for additional analysis to handle the heterogeneity between EAs.

**Table 2:** Results of Null Bayesian Multilevel Logistic Regression Model on factors associated with malaria under the age of 5 years old, Ethiopia, 2015

Fixed part	Estimate	SD	95% credible interval
β_0_(intercept)	−4.56	0.14	(−4.84–4.31)
Random part	Estimate	SD	95% credible interval
σ_*u*_^*2*^	0.19	0.15	(0.03 0.58)

When we compare the above two null models, even though, both of them suggested using multilevel analysis for additional analysis, the estimation of random effect shows a wide difference between the estimation of the classical analysis approach and the bayesian analysis approach. Based on classical estimation the random variance was 3.12 with an ICC of 0.49 whereas in Bayesian estimation it was 5.26 with an ICC of 0.62. Thus, the Bayesian multilevel random intercept model is more appropriate than the classical multilevel logistic regression in explaining the variation of malaria across enumeration areas in Ethiopia. Therefore, in this study multilevel analysis using the bayesian estimation technique was considered as a proper approach to estimate model parameters.

### Bayesian multilevel logistic regression analysis

The gibbs sampler procedure was applied in three different chains with 123,790 total iterations. After10000 burn-in terms discarded a total of 22,758 samples generated from the full posterior distribution. Non-informative normal prior distribution with mean = 0 and precision = 0.001 for the fixed effect and gamma distribution with scale = 0.1, shape = 0.1 for the variance of random effect was used. The convergence produced from markov chains has been confirmed by convergence assessment plots, before taking any inference from the posterior distribution.

### Assessment plots of model convergence

#### History plot

**Fig. 2:**
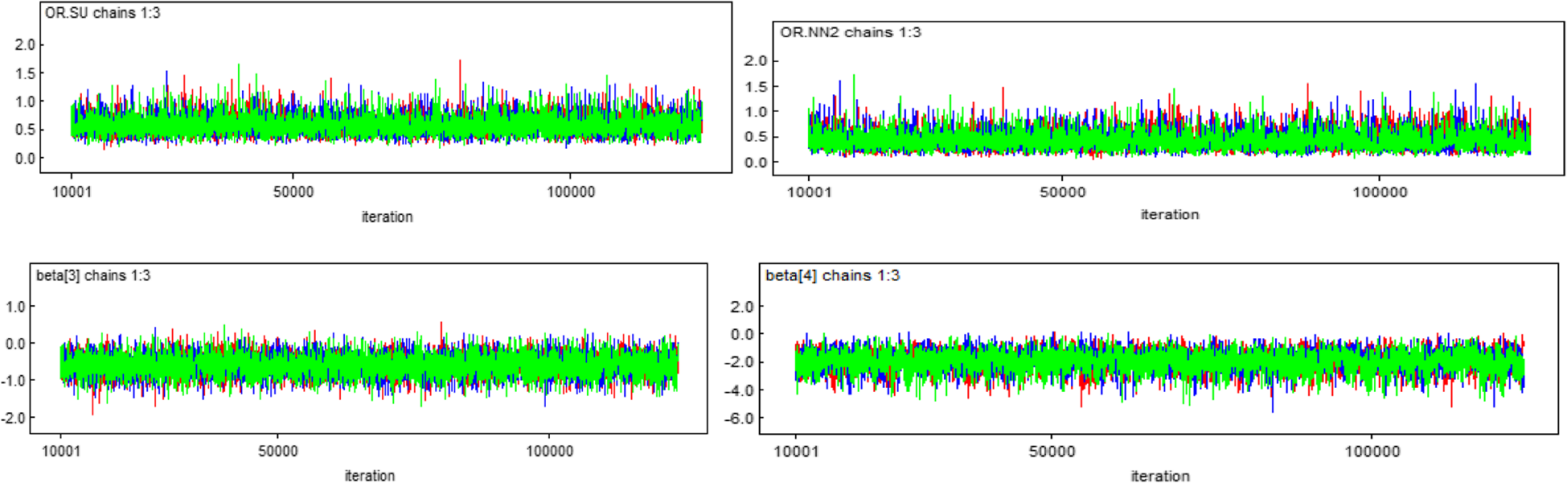
History plot for some of the significant parameters in the model

In this study, the three independently generated chains demonstrated good “chain mixture”, an indication of convergence (Fig. 2).

#### Kernel density plot

**Fig. 3:**
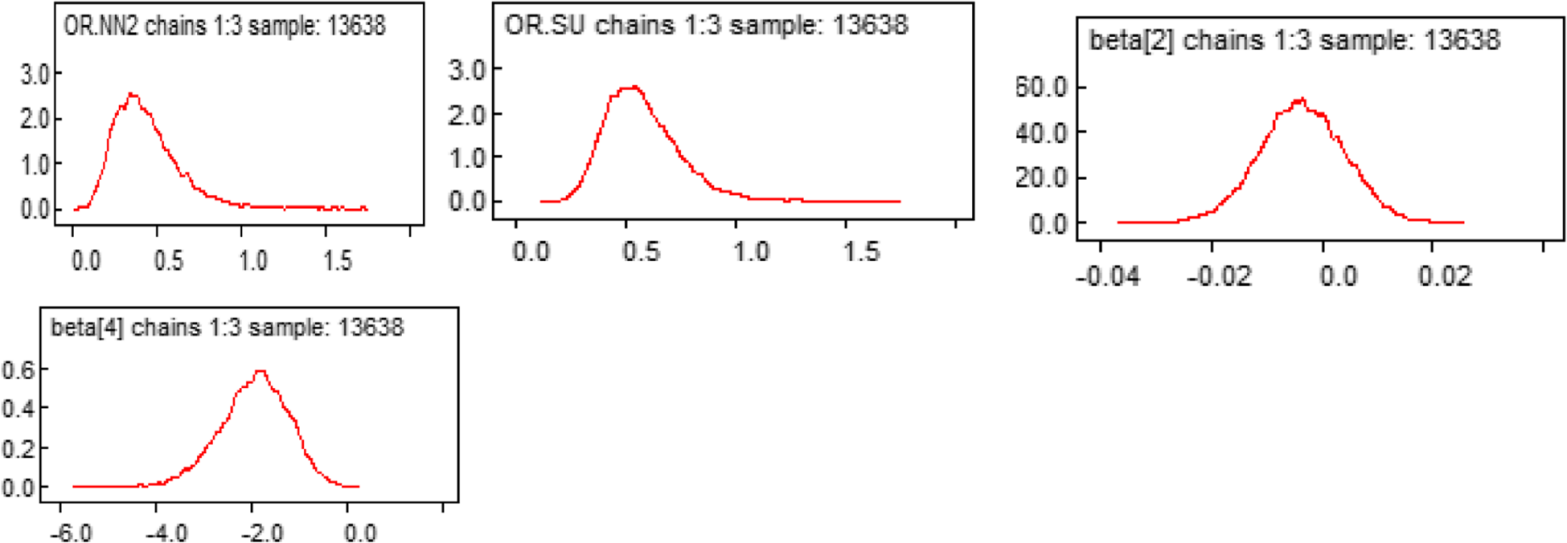
Density plot for some of the significant parameters in the model

The simulated samples from the posterior distribution for each regression coefficient are flat, the uni-modal shape of posterior marginal distribution indicating that simulated parameter value indicates convergence to the target distribution (Fig. 3).

#### Autocorrelation

**Fig. 4:**
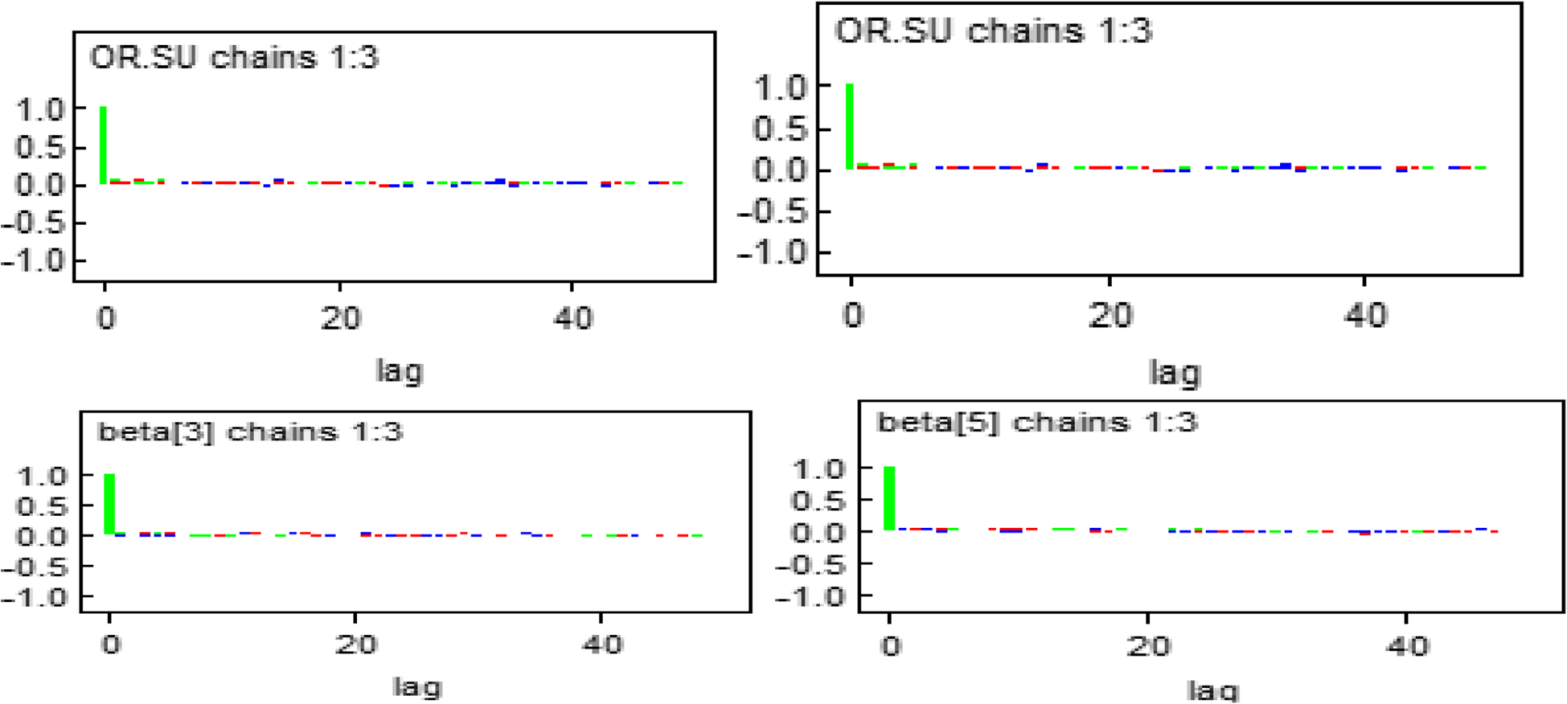
Autocorrelation plot for some of the significant parameters in the model

The plots show that the three independent chains were mixed or overlapped to each other, which indicates the presence of convergence (Fig. 4).

#### Gelman-Rubin statistic

**Fig. 5:**
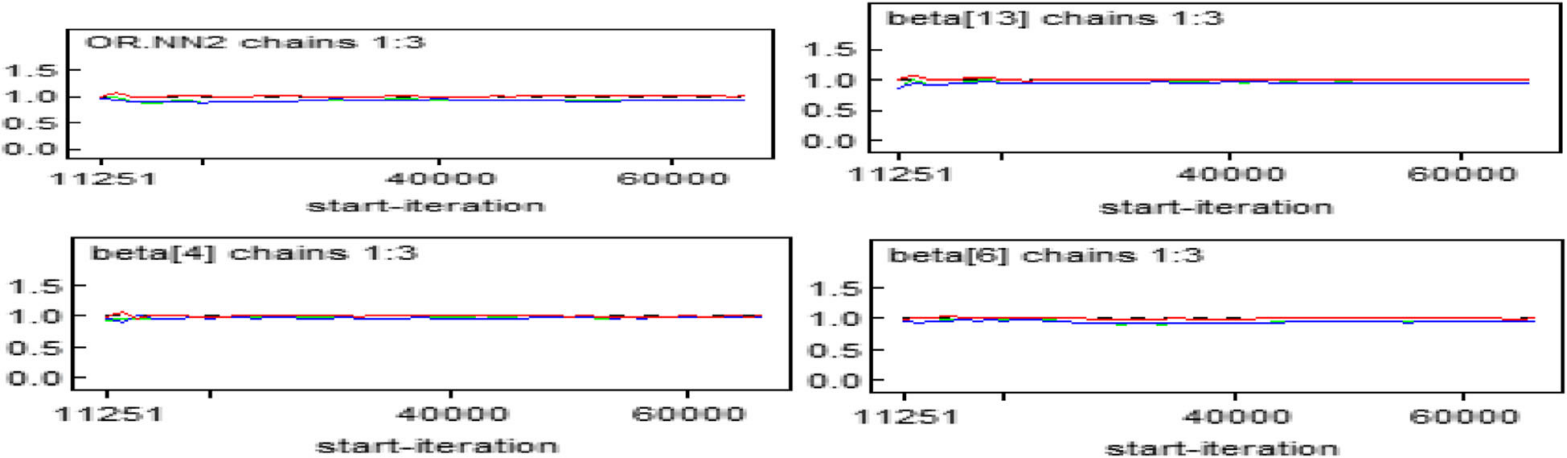
Gelman-Rubin Statistics plot for some of the significant parameters in the model

On the plots, the green line represents the between variability, the blue line represents the within variance and the red line represents the ratio. Hence, in this plot, the red line seems exactly on 1 which provided the evidence for convergence of parameters (Fig. 5).

### Model comparison and selection

**Table:** 

Models	DIC values
Null model (Model with no covariates)	802.53
A model with only individual-related variables	703.84
A model with community-related variables	770.89
Full model (model with individual and community-related variables)	654.02

Among the four models fitted, the full model has the smallest (654.02) DIC value. Therefore, the full model is most likely to fit the data.

### Bayesian multivariable multilevel logistic regression model

A Bayesian multivariable multilevel binary logistic regression model was fitted to identify the effect of explanatory variables. The parameters were estimated from the posterior distribution (Table 3).

**Table 3:** Posterior parameter estimates, SD, MC error, and odds ratios with their 95% credible intervals of the final model for factors associated with malaria under the age of 5 years old, Ethiopia, 2015

Variables	Categories	Estimate	SD	MC error	AOR	95% CI for AOR
Intercept		−1.37	1.744	0.086		
Age in months		−0.004	0.008	0.0001	0.99	0.98, 1.01
Sex	Female(ref)					
	Male	0.09	0.268	0.002	1.14	0.65, 1.86
Sleep undernet	No(ref)					
	Yes	−0.59	0.288	0.003	0.58	0.31, 0.97^a^
No of nets	None (ref)					
	One	−0.81	0.491	0.007	0.50	0.16, 1.08
	Two & more	−0.92	0.420	0.006	0.43	0.17, 0.88^a^
Have radio	No (ref)					
	Yes	−0.95	0.362	0.005	0.41	0.19, 0.78^a^
Have Electricity	No (ref)					
	Yes	−1.94	0.725	0.012	0.18	0.03, 1.52
Have Tv	No (ref)					
	Yes	−2.39	1.419	0.017	0.19	0.01, 0.89^a^
Roof material	Corrugate (ref)					
	Stick and mud	−0.01	0.541	0.006	1.15	0.34, 2.83
	Thatch	−1.04	0.393	0.005	0.38	0.16, 1.02
Floor Material	Cement Floor(ref)					
	Earth/Sand	−1.598	0.637	0.014	0.25	0.06, 1.01
	wood floor	−1.432	0.892	0.015	0.35	0.04, 1.39
Toilet facility	No facility (ref)					
	Pit latrine	−0.06	0.362	0.004	1.01	0.47, 1.94
	Latrine with flush	−0.20	0.735	0.007	1.06	0.18, 3.28
Interior Wall	No (ref)					
	Yes	−0.37	0.408	0.006	0.75	0.30, 1.47
Time to get Water	30 min&longer (ref)					
	less than 30 min	−0.40	0.339	0.005	0.71	0.34, 1.30
Water Source	Protected					
	Water (ref)	0.05	0.423	0.006	1.15	0.47, 2.47
	Tap Water	−0.01	0.439	0.005	1.09	0.42, 2.37
	Unprotected Water					
Altitude		−3.06	0.565	0.023	0.05	0.01, 0.13^a^

^a^Statistically significant variables at the 95% credible interval, *ref* reference

In the final model: sleeping under the insecticide-treated bed nets during bed time (ITN), number of ITN available in the household, altitude, availability of radio and television were statistically associated with malaria among under-five children at 95% credible interval.

Holding other covariates constant, the odds of having malaria for under-five-year-old children decreases by 95% with an increase in altitude (AOR 0.05, 95% CI 0.01–0.13).

After adjusted other covariates, children living in households with mosquito bed nets greater than two and more in their household were decreased the odds of having malaria by 57% than those in households without mosquito bed nets (AOR = 0.43, CI 0.17–0.88) and those children who were using mosquito bed nets were found to be decreased the risk of having malaria by 42% compared to those who were not using mosquito bed nets (AOR = 0.58, CI 0.31–0.97).

Keeping other covariates constant, children in households with television were decreased the risk of having malaria by 81% than those in households without television (AOR 0.19, 95% CI 0.01–0.89) and children in households with radio were decreased the odds of having malaria by 59% compared to those in households without radio (AOR 0.41, 95% CI 0.19–0.81).

## Discussion

This study aimed to investigate the relationship between the malaria status of children under the age of 5 years and socio-economic, demographic, and environmental factors based on the EMIS 2015 data using a bayesian analysis approach.

According to this study, the number of nets in the household, sleep under the net, altitude, availability of television and radio were statistically significant at 95% Bayesian credible interval.

Based on this study, among children residing in households with greater than two and more mosquito bed nets, the odds of having malaria was decreased by 57% compared to those in households without nets. This supports studies previously conducted in north-west Ethiopia, Nigeria, and north-east Tanzania [37–39].

According to the findings of this study, the use of a mosquito net has also been found as a protective factor against malaria cases among under-five children in Ethiopia. It revealed that those who were slept under mosquito bed net had a 42% decreased risk of malaria positivity as compared to those who did not use a bed net. This results supports also the study conducted by Hadya Zone, Southern Ethiopia which stated that those who were not using bed net were 4.67 times more likely to be infected [40] and another study conducted in Kenya also showed that malaria prevalence was observed to be more than two times higher among households that did not use mosquito nets compared to net user households [6]. This finding also consistent with studies done in different areas [21–23, 37, 38, 41]. This might be the fact that insecticides on the net repel mosquitoes, as a result, it decreases the number of mosquitos that enters into the house and attempts to feed on people inside. Besides this, ITN could also be a protective barrier and kill mosquitoes as well [42].

This study found that the availability of mass media played an important role in the prevention of malaria. Compared to children in households without television and radio, those in households with television and radio had 81 and 59% decreased risk of malaria positivity respectively. This finding is in agreement with the reports in Kenya, children of households with no access to television more likely to have malaria than their [6].This supports the positive findings of the influence of mass media for eliminating malaria in African settings [43]. This might be due to those who have watched television or listened to radio programs that might have information related to malaria prevention methods [6].

The association between malaria and altitude showed that the odds of malaria for children decreased with an increase in altitude. This is in agreement with the study conducted in southern Ruanda and Ghana [44, 45]. The finding also supports other studies conducted in Kenya, Uganda, and Northeastern Tanzania [6, 21, 37]. This might be due to the low altitude area is appropriate for mosquito breeding due to its flat topography that allows the collection of water following the rainy season [26].

Due to the fact this analysis was on a secondary data source, there is the possibility that some factors associated with malaria infection were not considered in this analysis because of dependence on variables collected only during the EMIS 2015 survey. Another limitation is that the survey used a cross-sectional design to collect data as such no causal inferences can be made between malaria infection and its determinants. Despite these limitations, the study used survey data collected from a nationally representative sample and has identified some of the predictors of malaria infection as such, the results can be generalized to determinants of malaria among under-five children in Ethiopia that can be used for planning future interventions for malaria prevention. Additional systematic review and meta-analysis study is recommended to have a pooled estimation of malaria prevalence and its determinants at the national level.

## Conclusion

The study revealed that sleeping under ITN, availability of ITNs for the household, altitude, availability of radio, and television for the household were the predictors of malaria among under-five children in Ethiopia. Thus, the government should strengthen the availability and utilization of ITN to halt under-five mortality due to malaria. Moreover, the government should also emphasis for the community living in low altitude areas and those residing households with less availability of television and radio.

## Data Availability

The datasets used and/or analyzed during the current study are available from the corresponding author on reasonable request.

## Abbreviations

ACIPH: Addis Continental Institute of Public Health
ACT: Artemisinin-based Combination Therapy
AOR: Adjusted Odds Ratio
ASL: Above Sea Level
CI: Credible Interval
CSA: Central Statistical Agency
DIC: Deviance Information Criteria
EA: Enumeration Area
EDHS: Ethiopian Demographic and Health Survey
EMIS: Ethiopia National Malaria Indicator Survey
EPHI: Ethiopian Public Health Institute
FMOH: Federal Ministry of Health
GDP: Growth Domestic Product
GPS: Global Positioning System
ICC: Intra-cluster Correlation Coefficient
IRS: Indoor Residual Spraying
ITN: Insecticide Treated Net
LLIN: Long-Lasting Insecticide-Treated Net
MCMC: Markov Chain Monte Carlo
MRC: Medical Research Council (Cambridge)
NSP: National Strategic Plan
PMI: President’s Malaria Initiative
RDT: Rapid Diagnostic Test
RBM: Roll Back Malaria
SD: Standard Deviation
SNNPR: Southern Nations, Nationalities, and Peoples’ Region
UK: United Kingdom
WHO: World Health Organization
